# The roles of glucose metabolic reprogramming in chemo- and radio-resistance

**DOI:** 10.1186/s13046-019-1214-z

**Published:** 2019-05-23

**Authors:** Jinguan Lin, Longzheng Xia, Jiaxin Liang, Yaqian Han, Heran Wang, Linda Oyang, Shiming Tan, Yutong Tian, Shan Rao, Xiaoyan Chen, Yanyan Tang, Min Su, Xia Luo, Ying Wang, Hui Wang, Yujuan Zhou, Qianjin Liao

**Affiliations:** The Affiliated Cancer Hospital of Xiangya School of Medicine, Central South University and Hunan Cancer Hospital, Key Laboratory of Translational Radiation Oncology, Hunan Province, 283 Tongzipo Road, Changsha, 410013 Hunan China

**Keywords:** Metabolic reprogramming, Chemo-resistance, Radio-resistance, TME

## Abstract

Reprogramming of cancer metabolism is a newly recognized hallmark of malignancy. The aberrant glucose metabolism is associated with dramatically increased bioenergetics, biosynthetic, and redox demands, which is vital to maintain rapid cell proliferation, tumor progression, and resistance to chemotherapy and radiation. When the glucose metabolism of cancer is rewiring, the characters of cancer will also occur corresponding changes to regulate the chemo- and radio-resistance of cancer. The procedure is involved in the alteration of many activities, such as the aberrant DNA repairing, enhanced autophagy, oxygen-deficient environment, and increasing exosomes secretions, etc. Targeting altered metabolic pathways related with the glucose metabolism has become a promising anti-cancer strategy. This review summarizes recent progress in our understanding of glucose metabolism in chemo- and radio-resistance malignancy, and highlights potential molecular targets and their inhibitors for cancer treatment.

## Background

Cancer is a serious public health problem. The incurrence and mortality is increasing year by year [1]. In addition to conventional radiotherapy, chemotherapy, and surgery, there are currently more and more popular neoadjuvant chemotherapy and molecular targeted therapies. These treatment options can cure early and part of the intermediate tumors in certain degrees, but are not ideal for most of cancer in middle and late stages [2]. Among many reasons, the treatment resistance is the one of major drawbacks. Radiotherapy and chemotherapy, as the routine treatment, face substantial challenges of resistance. However, the characters of chemo- and radio-resistance in different kinds of cancers are not exactly the same.

In the early 1920s, German biochemist and physiologist Otto Warburg conducted groundbreaking research and proposed the famous “Warburg effect”: Tumor cells prefer to use glycolysis for glucose metabolism even in oxygen-rich conditions, rather than more efficient mitochondrial oxidative phosphorylation for ATP production [3]. Actually, the entire metabolic network reprograms under the control of oncogenes and tumor suppressor genes, and the flow of nutrient in metabolic networks is also redefined in the process of tumorigenesis. Metabolic reprogramming provides critical information for clinical oncology. The aberrant glucose metabolism is a major kind of metabolic reprogramming in cancer [4], and recent studies have shown that aberrant glucose metabolism regulates cancer proliferation, cell cycle, drug resistance, and DNA repair [5–7]. As the molecular mechanisms underlying chemo- and radio-resistance are still poorly understood, the alteration of glucose metabolism in cancer provides new ideas to explain chemo- and radio-resistance. Herein, this review updates the mechanisms of metabolic reprogramming involved in tumor chemo- and radio-resistance.

## Main text

### The overview of glucose metabolic reprogramming

**Fig. 1:**
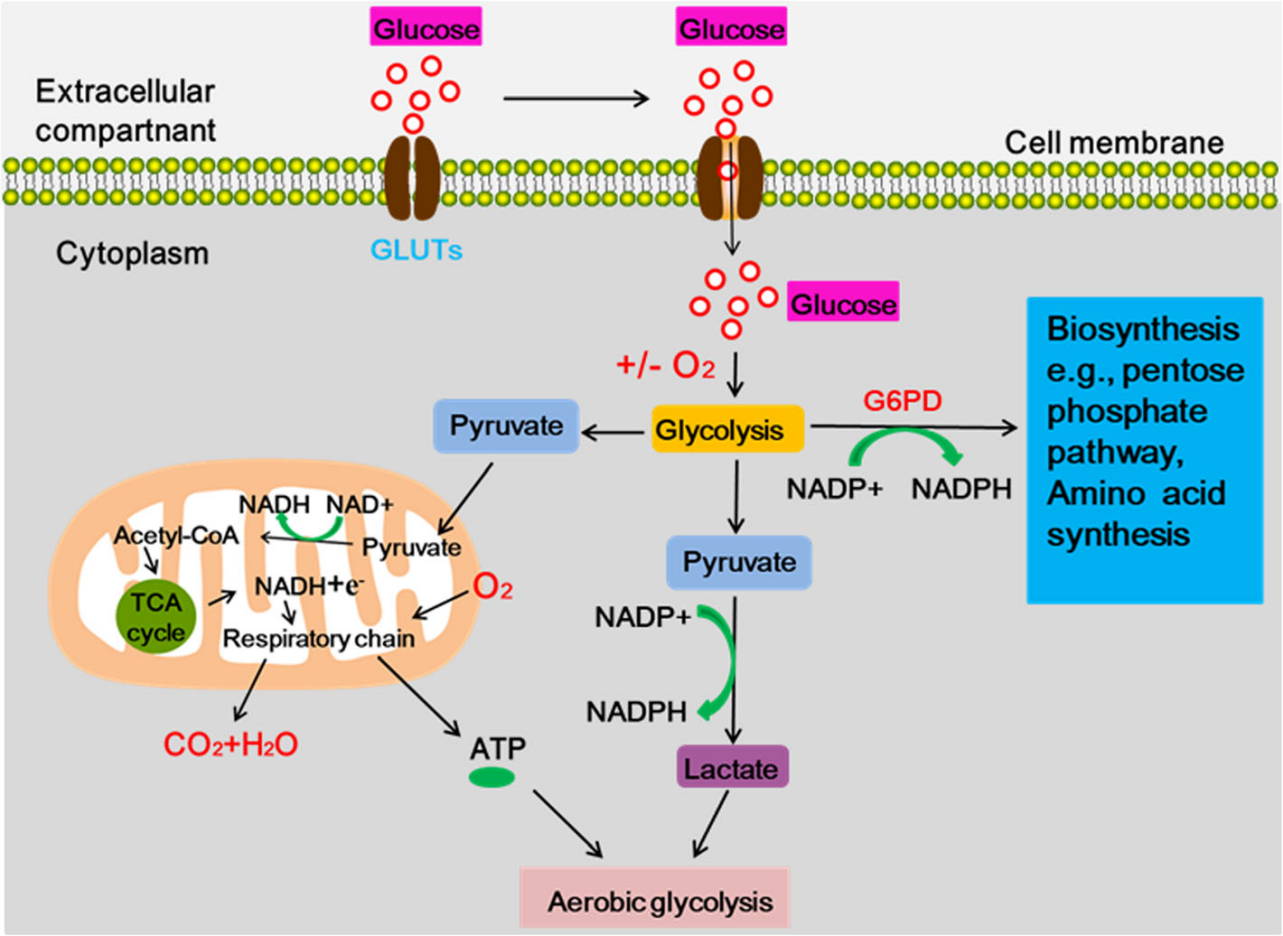
The energy metabolism of cancer cells. Under aerobic condition, Most of the glucose is first converted to pyruvate via glycolysis in the cytosol. Most pyruvate are mostly processed to lactate via glycolytic pyruvate even in the presence of oxygen, and only a small portion of pyruvates enters the mitochondria to produce CO_2_ by undergoing TCA cycle. In addition, small proportion of the glucose is diverted into the upstream of pyruvate production for biosynthesis (e.g., pentose phosphate pathway, and amino acid synthesis)

Metabolic reprogramming refers to the redefinition of the flow and flux of nutrient in tumor cells in the metabolic network to meet the needs of tumor cells for energy and anabolism [8]. Under oxygen-rich conditions, normal or differentiated cells can metabolize glucose and produce carbon dioxide through a tricarboxylic acid cycle (TCA), which produces 30 or 32 mol of adenosine triphosphate (ATP) per mole of glucose and a small amount of lactate during oxidative phosphorylation [9]. Only under hypoxic conditions, normal or differentiated cells produce large amounts of lactic acid by anaerobic glycolysis. However, German scientist Otto Warburg first proposed that tumor cells rely mainly on glycolysis to provide energy under aerobic conditions [3]**(**Fig. 1**)**. Weinberg characterized “aberrant metabolic phenotype” with “autologous proliferation signaling, apoptosis resistance, evasion of proliferation inhibition, continuous angiogenesis, infiltration and migration, unlimited replication capacity, immune escape” in tumor cells.

Glucose metabolic reprogramming between aerobic glycolysis and oxidative phosphorylation, previously speculated as exclusively observable in cancer cells, exists in various types of immune and stromal cells in many different pathological conditions other than cancer [6]. It has been well established that tumor cells have elevated rates of glucose uptake and high lactate production in the presence of oxygen, known as aerobic glycolysis (also termed the Warburg effect) [10]. As a matter of fact, high lactate production also remodels the tumor microenvironment (TME) by contributing to acidosis, acting as a cancer cell metabolic fuel and inducing immunosuppression resulting in aggressive proliferation, invasion, migration and resistance therapy [4]. However, the molecular mechanisms involved in the changes of glucose metabolism are complex. Changes in the tumor microenvironment, activation of oncogenes, and inactivation of tumor suppressor genes all contribute to the disruption of metabolism and steady-state metabolism of cells, ultimately leading to aberrant glucose metabolism [11, 12]. Specific oncogenes activation or tumor suppressor genes deactivation can reprogram the underlying metabolism of tumor tissues. Some genes can act as initiators of glucose consumption, include myc, KRAS, and BRCA1 [13–15]. Despite the progression, we still do not fully know the metabolic pathways that are reprogrammed by oncogenes or suppressor genes.

### Glucose metabolic reprogramming and chemo- and radio-resistance

Tumor cell survival under aberrant metabolism of glucose is a vital step not only for the process of tumorigenesis but also in treatment resistance and recurrence, especially for the occurrence of treatment resistance [4]. Chemotherapy in the form of neo-adjuvant or adjuvant therapy is the dominant treatment for most of cancers; the resistance directly affects the survival and prognosis of cancer patients [16]. Theoretically, the tumor mass, made of distinct chemo-resistant cell populations has been recognized as an important mechanism for chemo-resistance [17]. Actually, inhibition of glycolysis not merely inhibited cell proliferation, but alleviated resistance to chemotherapeutic drugs.

Existing evidence indicates that increased glucose uptake and enhanced aerobic glycolysis are able to induce the intrinsic or acquired resistance to DDP in gastric cancer cells [18]. Elevated lactate levels caused by aberrantly activated glycolysis can reinforce DNA repair and promote cisplatin-resistance in cervical carcinoma cells via the inactivation of histone deacetylase [19]. High-precision radiation therapy enables radiation oncologists to decrease delivery of an excessive dose of radiation to normal tissues and also to administer a high and booster dose of radiation, particularly to small target fractions in a malignant tumor [20]. Previous studies have revealed that the Warburg effect or aerobic glycolysis promotes the radio-resistance of various malignant tumors via generating a chemically reduced milieu associated with the development of radio-resistance in laryngeal carcinoma, prostate cancer, head and neck cancer [21–26]. For example, activation of adenosine monophosphate-activated protein kinase (AMPK) mediates metabolic reprogramming in resistant cancer cells through promoting both the Warburg effect and also mitochondrial biogenesis [27–30]. However, both the gene network triggering metabolic reprogramming and the molecular mechanism linking the reprogramming with radio-resistance remain to be determined.

### The mechanisms of glucose metabolic reprogramming-mediated chemo- and radio-resistance

Although increasing evidence has confirmed that glucose metabolic reprogramming can induce tumor radiotherapy and chemotherapy resistance, the specific mechanisms are still not clear [31–34]. The previously reported resistance mechanisms include mutations or increases in drug targets, changes in drug metabolism, and alterations in DNA repair, overexpression of anti-apoptotic genes, and inactivation of apoptotic gene products, immunosuppression and the formation of CSCs, etc.

With the increasing research understanding on the resistance of chemo- and radiotherapy, the researchers have pointed out that cancer stem cells, tumor microenvironment, autophagy, and exosomes are all closely related to tumor chemo- and radio-resistance. In fact, recent reports have shown that chemo- and radio-resistance acquisition is coupled to deregulate glucose metabolism and glycolysis [35]. Signaling pathways related to chemo-radiotherapy resistance are abnormally activated or inactivated during metabolic stress, such as Wnt, PI3K/AKT, Notch, NF-κB, MAPK [36–41]. In addition, the metabolic reprogramming mediated by aberrant expression of oncogenes can enhance the pentose phosphate pathway and aerobic glycolysis to promote the DNA repair and apoptosis resistance [42–44]. For example, the glucose metabolic reprogramming of colorectal cancer induced mainly by aberrant MYC expression could activate the pentose phosphate pathway, purine/pyrimidine synthesis pathway, fatty acid oxidation pathway and mitogen-activated protein kinase (MAPK) signaling pathway to prolong the survival of cancer cells under the chemotherapy and radiotherapy [45–47]. In truth, the metabolic reprogramming may induce the DNA repair, the immunosuppression of tumor microenvironment, the anti-apoptosis by enhanced autophagy, and the formation of cancer stem cells mediated by exosomes, which all induce chemo- and radio-resistance. Herein we will introduce mechanisms of glucose metabolic reprogramming in radiotherapy and chemotherapy resistance.

#### Activating DNA damage repair

**Fig. 2:**
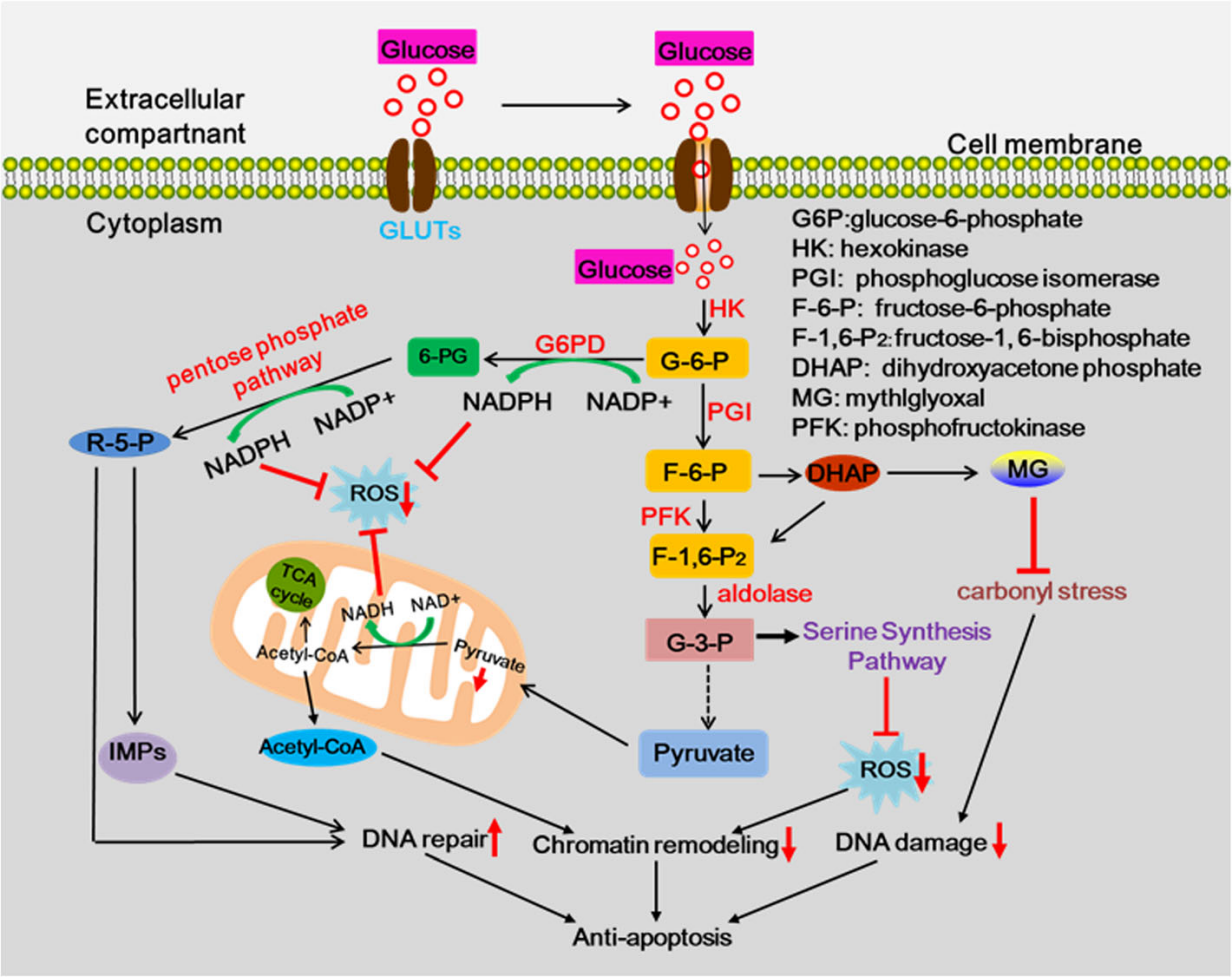
Simplified diagram of the main metabolic pathways involved in DNA damage/repair. Continuous activation of aerobic glycolysis can increase the capture of glucose into the cytoplasm by up-regulating the expression of glucose transporters (GLUTs) and substantially enhance the high rate of glucose influx via activating HK, PFK, and aldolase enzyme and promoting their expression, which in turn facilitates the aerobic glycolysis. The glycolytic switch in tumor cells allows the direct or indirect flux of glycolytic intermediates to many biosynthetic pathways (e.g., pentose phosphate pathway, serine synthesis pathway, MG pathway, and nucleotide synthesis), which provides the biomacromolecules and other materials required for prolonging the cancer cell survival via enhancing DNA repair, inhibiting DNA damage and decreasing chromatin remodeling

It’s well known that the essence of chemotherapy and radiotherapy is to cause the disruption of DNA replication, thus leading to cell death or apoptosis and achieving therapeutic purposes [48]. Accumulating evidence suggests that the continuous activation of aerobic glycolysis plays a vital role in tumor development and the expression of many altered genes is accompanied by aerobic glycolysis in tumor development and resistance [49, 50]. Efficient DNA damage repair would depend on anabolic alterations that could provide cancer cells with nucleotide pools for repair of radiation and chemotherapy-induced DNA damage [51]. Recent study has indicated that the chemo-resistant breast cancer cells and mesothelioma cells have high levels of aldehyde dehydrogenase (ALDH) activity. ALDH is an important detoxifying enzyme of glycolysis, which belongs to a class of detoxifying enzymes whose expression is linked to cancer chemo-resistance [52]. Meanwhile, glycolysis can also enable cancer cells to reduce the level of intracellular reactive oxygen species (ROS) by limiting the pyruvate flux into mitochondrial respiration, and thus acquire resistance to apoptosis and DNA impair**(**Fig. 2**)** [53–55].

An elevated endogenous ROS level generated from attacks of mitochondria on nearby mitochondrial DNA (mtDNA) results in an imbalance between production and destruction of ROS, which resulted in oxidative damage to mtDNA under aberrant condition of glucose metabolism [56–59]. ROS, which can increase oxidative DNA damage and hence the load of the DNA-repair machinery, are regulated through different metabolic pathways. High ROS levels affect many aspects of tumor biology like DNA damage and genomic instability. Furthermore, mutations in genes involved in the glucose metabolism rewiring can also block the balance of DDR (DNA damage response)and DNA repair to result in resistance to chemotherapy and radiotherapy. For example, PFKFB3 (6-phosphofructo-2-kinase/fructose-2,6-bisphosphatase 3), an altered genes significantly accelerates the glycolysis, enhances the ability of DNA repair and its pro-tumor effects during glycolysis through the PFKFB3/Akt/ERCC1 signaling pathway, thus leading to failure of chemotherapy and radiotherapy in HCC [60]. Furthermore, a study indicated that disrupting cancer metabolism had an important role for both glycolysis and glutaminolysis in promoting DNA-DSB (double strand breaks) repair and preventing accelerated senescence after irradiation [61].

The aberrant glycolysis and glutaminolysis could promote DNA repair via targeting the hexosamine biosynthetic pathway (HBP) and tricarboxylic acid (TCA) cycle [62]. The previous researches had indicated that Mucin1 (MUC1), an oncogene overexpressed in multiple solid tumors, can mediate DNA repair in breast cancer cells and facilitate the metabolic reprogramming in pancreatic cancer cells [63]. In MUC1-expressing pancreatic cancer cells, the metabolite levels in glycolysis, PPP and nucleotide biosynthetic pathways increased to enhance the DNA damage repair and inhibit the sensibility of radiation therapy and chemotherapy [64–67]. Furthermore, amplified N-Myc can directly bind with the tetrameric form of p53 at the C-terminal domain in the nucleus to alter p53-dependent transcriptional responses in neuroblastoma patients with wild-type p53, but wild-type p53 negatively regulates G6PD activity, a rate-limiting enzyme of the pentose phosphate pathway that is the most important sources of nucleotides, and then decreases dNTP synthesis, ultimately influencing the DNA repair [46, 68, 69]. Therefore, N-Myc directly suppresses the transcriptional responses of wild-type p53 to inhibit the pentose phosphate pathway and increase the DNA repair.

In essence, the DNA damage repair induced by glucose metabolic reprogramming is a complicated procedure which involves the activation of many oncogenes and activation or silencing of signaling pathways and needs more researches to clarify it.

#### The apoptosis resistance of enhanced autophagy

Autophagy is an evolutionarily conserved process through which lysosomal degradation of damaged and superfluous cell components are recycled back into basic biomolecules in the cytosol [70, 71]. Low glucose levels could induce autophagy in a wide variety of mammalian cell types, including cancer cells, and this regulation appears to be partially dependent on the activation of AMPK [72]. Enhanced autophagic activity buffers glucose and amino acid starvation most likely by degrading intracellular energy reserves like glycogen, and proteins [73]. E.g. CAFs (cancer-associated fibroblasts) in the tumor stroma exhibit robust activity in terms of aerobic glycolysis and autophagy due to loss of caveolin 1 (Cav-1) expression [74–77]. CAFs with higher levels of aerobic glycolysis and autophagy in the tumor stroma can produce more IL-8 and activate the NF-κB signaling pathway, ultimately leading to resistance to cisplatin in human gastric cancer [75, 76, 78]. In general, enhanced autophagy protects cancer cells during chemotherapy and radiotherapy via supporting the survival of tumor cells, leading to cancer resistance and refractory cancer [75, 79–83]. In addition, increased autophagy regulated by the PI3K/AKT/mTOR pathway prolongs cancer cell survival via resisting to apoptosis under acidic environment stress produced by glycolysis [84].

A new study has found that autophagy is a major way of down-regulating cell metabolism, leading to cancer cell quiescence, survival, and chemo-resistance [85, 86]. The up-regulation of autophagy mediated by metabolic dysfunction could contribute to a common mechanism of resistance to chemotherapy and radiotherapy by suppressing apoptosis, such as rapamycin (Rp) [87–89]. In addition, the induction of autophagy may defend against epirubicin-mediated apoptosis, act as a pro-survival factor, and thus lead to deficient apoptosis in HepG2 and A549 cells [90–92]. Besides, a great deal of evidence suggests that autophagy mostly causes cancer cell survival and resistance to treatment through activation of different autophagy-associated molecules and signaling pathways, such as Wnt, PI3K/AKT, Notch [93–95]. Whereas, autophagy inhibition could promote tumor cell death and enhance the sensibility of radio- and chemotherapies [4, 92, 96–98]. Most studies have suggested that autophagy promotes chemoresistance and targeting autophagy-associated molecules may increase cancer cell chemo-sensitivity [99]. An up-regulation of autophagy may represent a mechanism of resistance to oxidative stress induced by chemotherapeutic drugs and may potentiate the survival to hypoxia and nutrient starvation resulting from the frequently defective tumor vascularization [100]. For example, induction of p53 and transfection of ERK activating RAS mutants but not AKT activating RAS mutant in p53-null ovarian cancer cells promoted autophagy, although the autophagy induced by p53 or ERK activating RAS mutants showed an opposite sensitivity to cisplatin treatment because the activation of RAS/ERK ultimately lead to the increased expression of p-ERK and Bcl-2 and the decreased expression of p-AKT and Bax [101]. Furthermore, a recent study showed that HK-2 (hexokinase-2), a key enzyme of the rate-limiting step in glycolysis up-regulates cisplatin-resistance in ovarian cancer cells by enhancing cisplatin-induced autophagy [102]. Whereas, decreased autophagy induced by Baf A1 treatment, a pharmacological autophagy inhibitor, and knockdown of ATG5 that blocks the non-selective macroautophagy pathway significantly increased apoptotic cell death in chemoresistant breast cancer cells [103]. In the chemo-resistant and radio-resistant cancer cells under periods of glucose metabolic stress, the increased autophagy could prevent cancer cells from apoptosis induced by ER stress (endoplasmic reticulum stress) [104]. As a kind of autophagy, moreover, the enhanced mitochondrial autophagy can prevent apoptosis by reducing mitochondrial outer membrane permeability (MOMP) and reducing the release of mitochondrial pro-apoptotic proteins, such as cytochrome C and SMAC/DIABLO [105].

In spite of a spur in research articles demonstrating the role of autophagy in cancer, the exact role of autophagy induced by metabolic reprogramming on tumor cells is still controversial and remains to be further elucidated [106]. Many of the pathways that control autophagy are deregulated in cancer, and cancer therapeutics targeting these pathways activates autophagy. Taken together, the role of autophagy in tumor initiation and drug resistance is likely context-specific. The functional role of autophagy in these settings needs to be established. A particularly interesting possibility is that autophagy favors tumor cell survival. If this is correct, then inhibition of autophagy might synergize with existing cancer treatments.

#### The immunosuppressive effect of tumor microenvironment

**Fig. 3:**
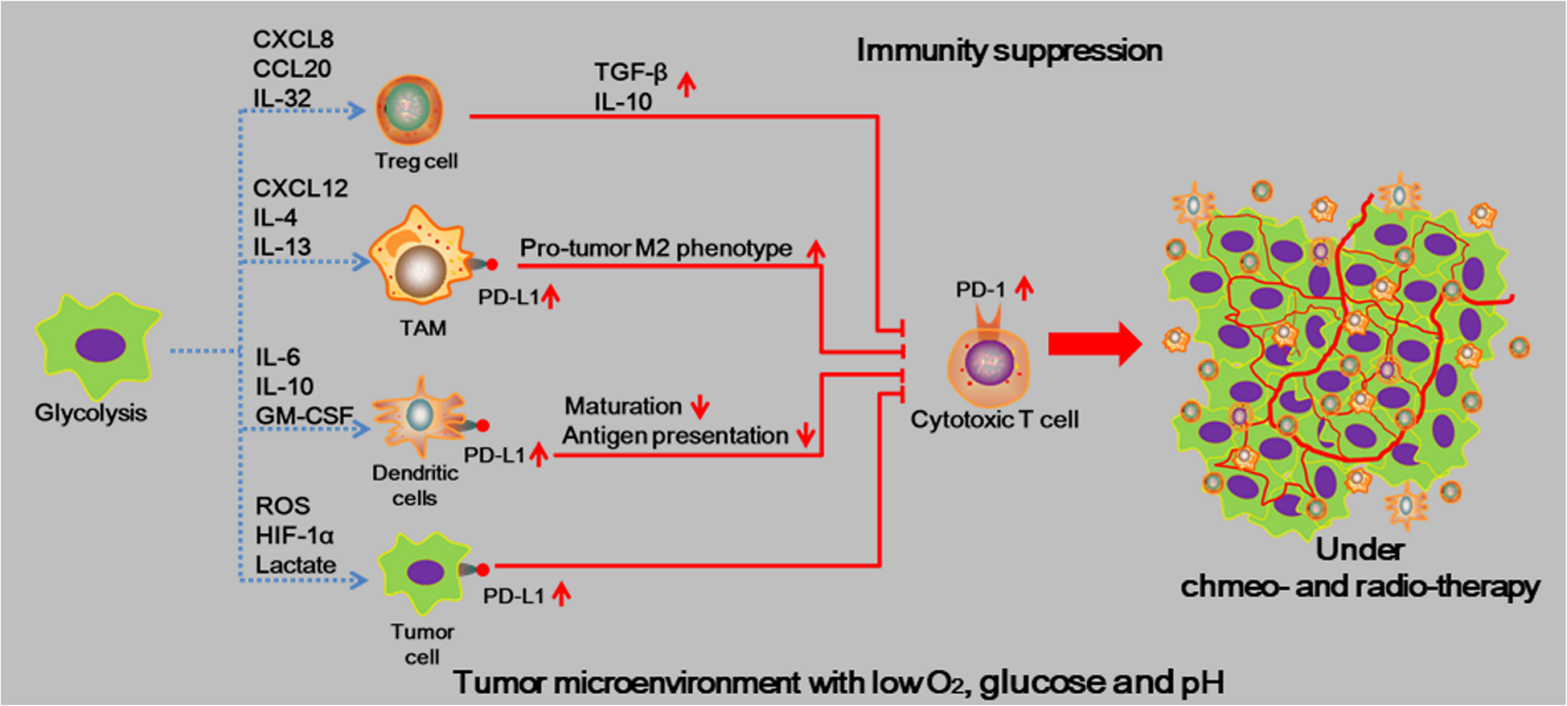
The immunosuppressive effect of the tumor microenvironment. The hypoxia and acidosis of the tumor microenvironment (TME) contribute to immunosuppression via several mechanisms. These mechanisms include increased accumulation, activation, and expansion of immunosuppressive regulatory T (Treg) cells; recruitment of inflammatory monocytes and tumor-associated macrophages (TAMs) and reprogramming of TAMs towards the pro-tumor M_2_ phenotype; suppression of dendritic cell (DC) maturation, which results in inhibiting activation of tumour-specific cytotoxic T lymphocytes (CTLs). Importantly, the programmed cell death protein 1 (PD-1)–programmed cell death 1 ligand 1 (PD-L1) pathway is often activated in the TME as a mechanism to evade anticancer immune responses, with up-regulation of PD-L1 expression on TAMs, DCs, and tumor cells. In addition, tumor-infiltrating CTLs typically up-regulate PD-1, limiting their cytotoxic potential against tumor cells. CCL20, C-C-motif chemokine ligand 20; CXCL, C-X-C-motif chemokine ligand; GM-CSF, granulocyte–macrophage colony-stimulating factor; TGFβ, transforming growth factor β; IL, Interleukin

Hitherto, as to metabolic reprogramming, tumor cells finely regulate ATP synthesis by regulating substrate uptake, as well as enzymes related to glycolysis, which enables them adapt to the nutrient microenvironment [107–112]. Metabolic changes occur not only in tumor cells, but also in immune cells infiltrated in the tumor tissues that undergo metabolic reprogramming to accommodate functional changes [113]. In fact, the altered tumor microenvironment (TME) can induce the tumor cells secretion of immunosuppressive cytokines to inhibit the immune effector cells or induction of suppressive immune cells to exert immunosuppressive effects, then inducing the immune escaping of cancer cells and ultimately contributing to chemotherapy and radiation resistance [114, 115]. During recent years, the interaction between immunosuppression and treatment resistance in different subsets of tumor cells within the TME was increasingly valued by cancer researchers [116–118] **(**Fig. 3**)**.

Tumor cells have to adapt their metabolism to survive and proliferate in this harsh microenvironment. Changes in the tumor microenvironment can affect the levels of infiltrating cell-associated chemokines in tumor cells. These chemokines, in turn, recruit Tregs to tumor tissues to exert immunosuppressive effects [119]. For example, under an inflamed microenvironment, the TLR (Toll-like receptor) can increase glucose uptake and lactate production in Treg cells through up-regulating the expression of key enzymes Glut1 (a glucose transporters), which is beneficial to the proliferation of Treg cells [102, 120]. Tregs exert immunosuppressive effects by inhibiting effector T cells and dendritic cells to enhance the effect of anti-apoptosis and the survival of cancer cells [121]. Because of the TME comprising of stromal and various components of the immune system where reprogramming of the metabolism manifests Warburg phenotype (enhanced aerobic glycolysis), it can play a significant role in suppressing the immune attack on the tumor cells leading to cancer cell survival, proliferation and resistance to therapies [122]. Moreover, Verduzco and others widely accept that the alterations in tumor microenvironment during chemo−/radiotherapy lead to the expression of TME-related factors, which significantly contributes to chemo−/radio-resistance [123–125]. E.g. Genetic ablation of AMPK activates mammalian target of rapamycin (mTOR) signal with enhanced expression of hypoxia-inducible factor-1 alpha (HIF-1α), resulting in rapid cellular proliferation accompanied by activation of aerobic glycolysis [29, 30, 126]. HIF-1α, a biomarker of the hypoxia microenvironment, demonstrates an emerging role in increasing resistance to current cancer therapies, including chemo−/radio-resistance [125]. Moreover, HIF-1α stabilized by hypoxia microenvironment is also able to activate the expression of PD-L1 by binding of HIF to a specific hypoxic response element in the promoter of PD-L1 in cancer cells [127, 128]. PD-L1 expression in cancer cells enables them to deliver an inhibitory signal to PD-1-positive T-cells, suppressing T-cell function. This may be responsible for the accumulation and the activation of immunosuppressive cells [129–131]. In addition, the under hypoxic condition, the tumor cells tend to be anaerobic with glucose and secrete IL-10 that thriggers STAT3 phosphorylation and activation of the PD-1/PD-L1 pathway [132]. In multiple myeloma (MM), increased glucose metabolism of cancer cells can increase the expression of HK-2 and lactate dehydrogenase A (LDHA) to reduce the therapeutic effects of standard care drugs, such as bortezomib and melphalan [133] via inhibiting T cell immunity and promoting cancer stem-like properties. Moreover, tumor LDHA affects MDSCs (myeloid-derived suppressor cells) to control tumor immunity [134]. Human MDSCs induced by granulocyte colony-stimulating factor (G-CSF) and granulocyte macrophage colony-stimulating factor (GM-CSF) inhibit T cell immunity in the tumor microenvironment in patients with cancer [135]. This strongly suggests the importance of cancer metabolic reprogramming in maintaining the interaction between the tumor microenvironment and the immunosuppression.

Regardless of the role of complexity components of TME in chemo−/radio-resistance of cancer cell, the concrete mechanisms of immunosuppression regulated by TME are still not verified and need lots of studies to confirm.

#### The formation of cancer stem cells mediated by exosomes

**Fig. 4:**
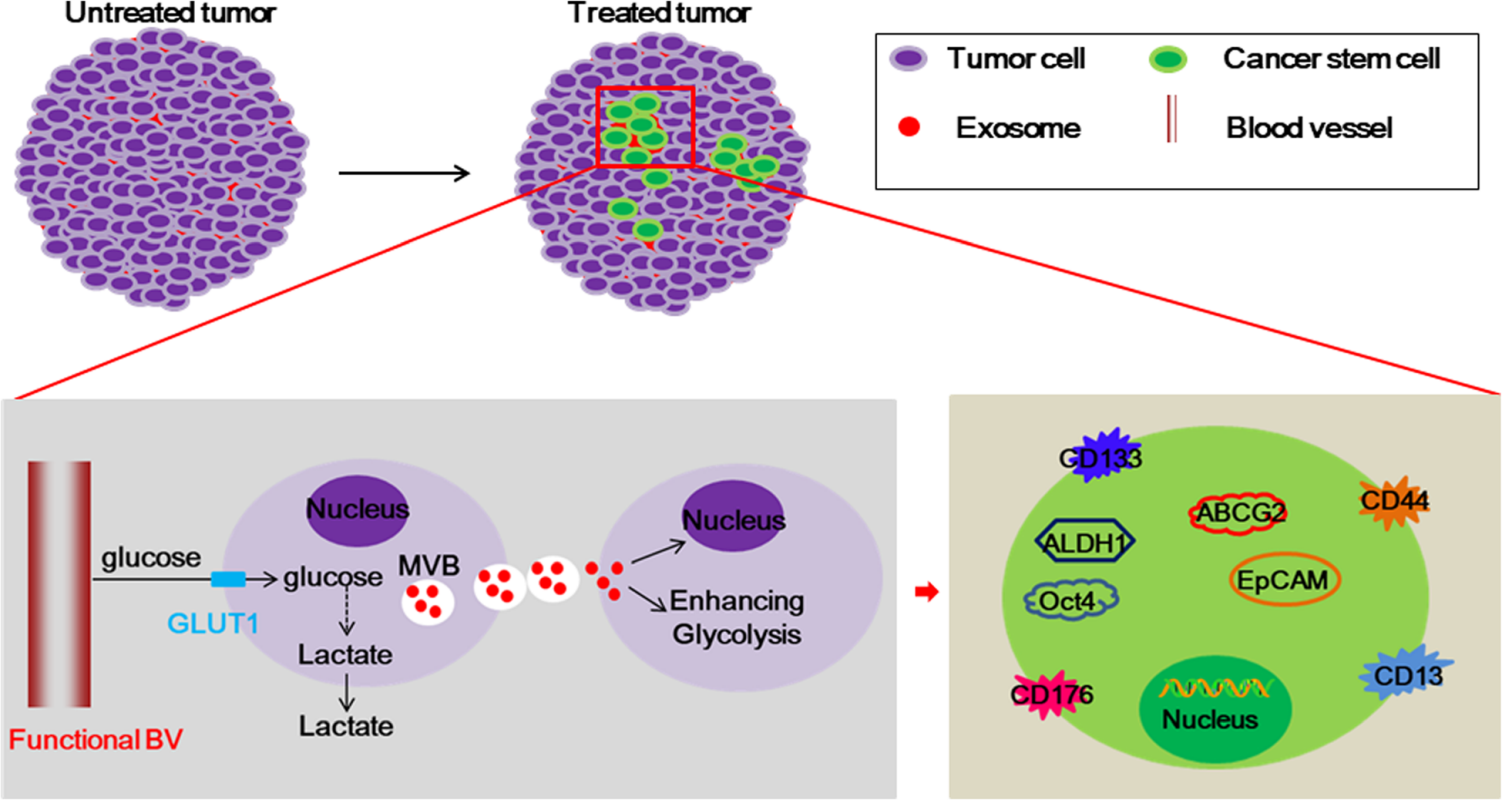
The role of the exosomes in the formation of CSCs. The cancer cells with enhanced glycolysis could release a large amount of exosomes contained several of glycolytic enzymes and CSCs markers. These exosomes can be taken up by the recipient cancer cells, and then promote the glycolysis and induce the dedifferentiation of cancer cells to acquire stemness phenotype through transfer their stemness-related molecules

Exosomes are 30–150 nm in diameter microvesicles derived from the multi-vesicular endosome pathway [136]. Cancer cells that utilize aerobic glycolysis as the main energy generating pathway can enhance the exosome secretion [137–140]. The increased secretion of endogenous exosomes from the resistant cancer cells can be taken up by recipient cells and leads to the modulation of aerobic glycolysis and chemotherapy and radiotherapy sensitivity [141–144]. For example, PC-derived exosomes (isolated from murine pancreatic cancer cells) could inhibit glucose intake and promote lipidosis, developing an eventual state of insulin resistance in skeletal muscle cells [142]. The newest documents have found that the exosomes can induce the formation of cancer stem cells (CSCs) to decrease the effect of chemo- and radio-therapy [145–147] **(**Fig. 4**)**.

The aberrant glycolytic reaction of CSCs contributes to therapy resistance via preserving stemness and tumorigenic properties of CSCs [148–150]. Exosomal LMP1 activates the PI3K/AKT pathway, and then up-regulates the expression of the surface marker CD44^+/High^, ultimately increasing the populations of CD44^+/High^ cells, which are the putative stem cell in nasopharyngeal carcinoma cells [150–152]. Besides, exosomal LMP1 could reduce the phosphorylation of AMPK and changed its subcellular location after irradiation, which appears to occur through a disruption of the physical interaction between AMPK and DNA-PK, and then causes decreasing in AMPK activity which is associated with LMP1-mediated glycolysis and resistance to apoptosis induced by irradiation [126, 153, 154]. Similarity, the resistant cancer cells with enhancing glycolysis can secrete a large amount of exosomes containing EpCAM protein, an epithelial cancer stem-like cell markers and glycolysis enzymes [126, 155–159]. The neighboring non-resistant cells can take up these exosomes and positively regulate mTOR and epithelial growth factor receptor (EGFR) signaling pathways to enhance the glycolysis and promote EpCAM^+^ tumor cells to ovarian cancer stem cells (CD133^+^ and CD117^+^CD44^+^) and putative drug-resistant tumor cell phenotype (EpCAM^+^ CD45^+^) transformation [152, 155, 159–162]. Besides, the exosomes secreted from resistant tumor cells can be taken up by non-resistant cells and induce the production of ROS via enhancing metabolic reprogramming [163]. The increased level of ROS can activate the Wnt signaling pathway to accumulate the cancer stem-like cells with CD44v8-10^high^/Fbw7^high^/c-Myc^low^ or CD44v8-10^high^/Fbw7^low^/c-Myc^high^, leading to the formation of resistant sites [147, 149, 152, 164].

Transport of exosomal components can contribute to the chemo- and radio-resistance of cancer cells [165–167]. Among them, transfer of miR-100, miR-222 and miR-30a from the exosomes derived from adriamycin- and docetaxel-resistant MCF-7 breast cancer cells to drug sensitive MCF-7 cells increased the drug resistance of the sensitive cell line through increasing CSCs proportion in cancer cell populations and promoting the phenotypic transition of non-CSCs toward the CSCs phenotype [168–170]. Actually, exosomal HSPs could be involved in the occurrence of EMT and ECM remodeling which were closely associated with the formation of stem cells to mediate the resistance of cancer cells [171]. E.g. exosomal HspDNAJB8, an Hsp40 family member, has a role in maintenance of renal cell carcinoma CSCs/CICs (called cancer stem–like cells/cancer-initiating cells), resistance to chemotherapy and radiotherapy [172, 173]. Similarly, the exosomal lncRNA UCA1 is demonstrated to possibly activate the Wnt signaling pathway and facilitate the malignant transformation of stem cell through the modification of the gene network by tail modification of histone to increase chemo-resistance of cancer cells [174, 175].

Exosomes are speculated as a novel target for solving the radio- and chemo-resistance because they can promote CSCs phenotype. However, the research about the role of exosomes in the treatment resistance of cancer is not much more; it isn’t a good explanation to verify the concrete effect of exosomes and need to more studies to confirm.

### Perspectives of metabolic inhibitors

Up to date, the metabolic inhibitors aim to inhibiting the enzymes about tumor metabolism, and then decrease the level of cancer glucose consumption to decrease amount of ATP, attenuating amino acids and nucleotides synthesis, and generate reactive oxygen species (ROS) [126, 176–182]. Metabolic inhibitors reduce the metabolite levels in glycolysis, PPP and nucleotide biosynthetic pathways to down-regulate the resistant effect of cancer cells via preventing DNA damage repair and enhancing chemotherapy and radiation responsiveness [47, 183]. For example, 3-BrPA (3-bromopyruvate), a special inhibitor of HK-2 kinase, can induce the imbalance of intracellular redox via inhibiting the glycolysis and strengthening the tricarboxylic acid cycle in cancer cells, during which a large amount of ROS is produced and accumulated in the cancer cells, destroying the normal structure inside the cell and causing the cell to gradually die [184]. Therefore, 3-BrPA can sensitize first-line anti-tumor drugs in the resistant cancer cells, such as 5-fluorouracil, doxorubicin, mycin, mitoxantrone and platinum drugs (e.g. cisplatin, oxaliplatin) [185]. In addition, the covalent inhibitor JX06 targeting PDK via structural modification hinders access of ATP to its binding pocket and in turn impairs PDK1 enzymatic activity, which increases the sensitivity of chemotherapy and radiotherapy by promoting cellular oxidative stress and apoptosis [186]. FX11, an LDHA inhibitor, can be capable of blocking aerobic glycolysis via inactivating the CK2/PKM2/LDHA axis to induce oxidative stress, and suppress drug resistance in various cancers [187]. 3PO, a glycolysis inhibitor targeting PFKFB3, can inhibit the glycolysis of nintedanib- and sunitinib-resistant tumor cells via inducing cell-cycle arrest and apoptosis, and thus promote the therapeutic efficacy of chemo- and radio-therapy [188].

Even though some metabolic inhibitors have been approved for clinical treatment, the efficacy is not ideal and rigorous evidence-based medical evidence lacks. There are inextricable links between cell metabolism, tumor immunity, and tumor epigenetics. Metabolic inhibitors can only achieve maximum biological efficacy when combined with targeted inhibitors of macromolecule synthesis, cellular immune-agonists, and agonists or inhibitors associated with metabolic pathways. Furthermore, most metabolic inhibitors lack specificity and cannot target tumor cells and have a killing effect on normal cells. Therefore, the researches on metabolic inhibitors have promising development prospects.

## Conclusions

**Fig. 5:**
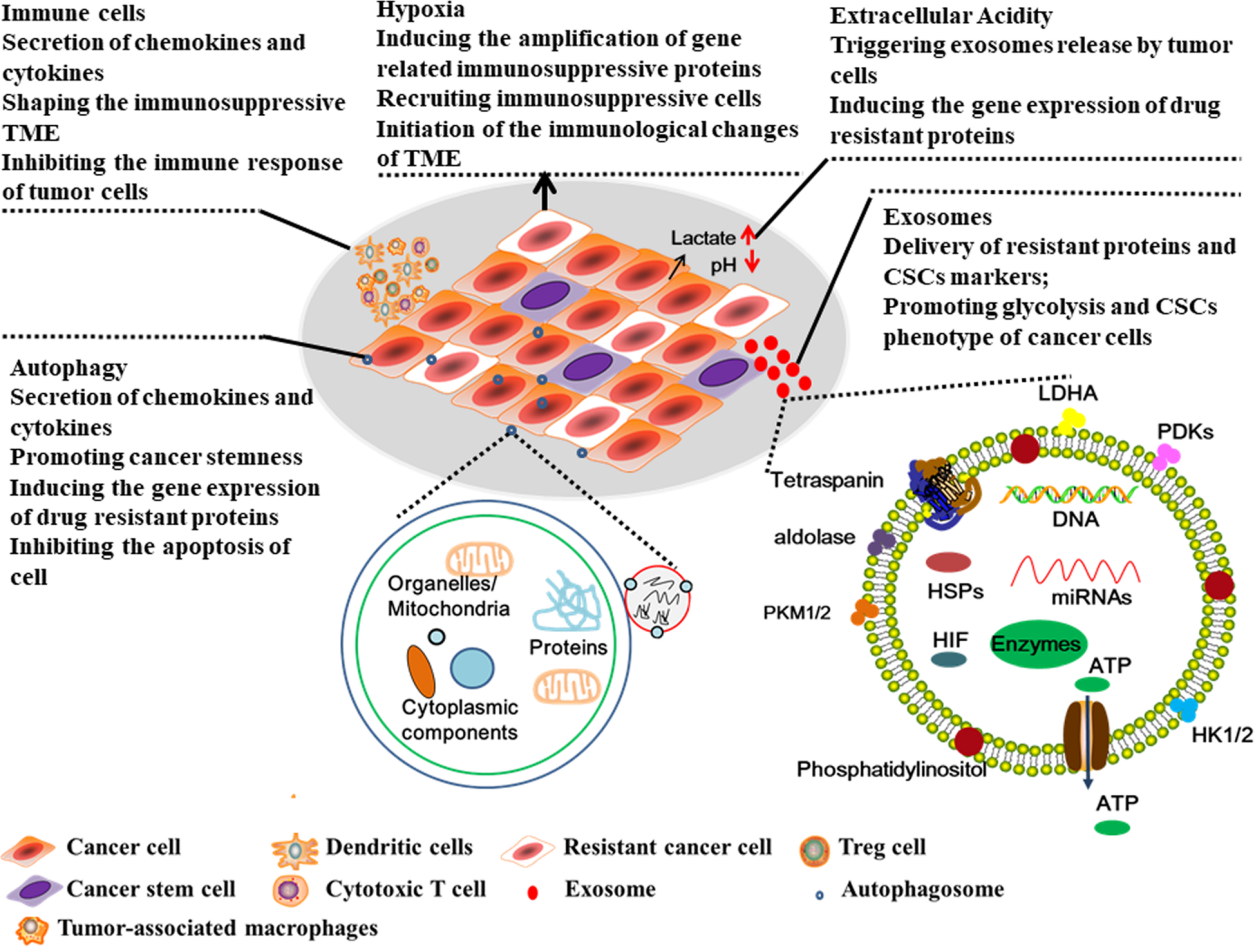
The overview of acquired chemoradiotherapy resistance mediated by metabolic reprogramming in cancer cells

Extensive studies have provided strong evidence for reprogramming of cancer metabolism in chemo- and radio-resistant cancer. Aberrant glucose metabolism could alter many physiological activities**(**Fig. 5**)**, e.g. inducing DNA damage repair, enhancing autophagy, changing tumor microenvironment and increasing the secretion of exosomes, etc. However, these alterations are not a simple relationship between chemo- and radio-resistance and glucose metabolism. Additional studies are needed to better understand the molecular mechanisms linking resistance to cell metabolism. Additionally, it will be important to understand whether the effects of metabolic inhibitors are cell type-specific. Because changes in treatment resistance can directly or indirectly impact multiple processes--including metabolism, ROS signaling, and calcium signals. The outcome may be critically dependent on cell types. Finally, once the interconnections between the glucose metabolism of cancer cells and resistance to treatments are better understood, we will hopefully be able to harness this information to devise therapies for cancer resistance.

## Abbreviations

6PGD: 6-phosphogluconate dehydrogenase
ALDH: Aldehyde dehydrogenase
AMPK: AMP-activated protein kinase
ATP: Adenosine triphosphate
CSCs: Cancer stem cells
DDR: DNA damage response
DNA-DSB: DNA-double strand breaks
EOC: Epithelial ovarian cancer
ETC: Electron transport chain
G6PD: Glucose-6-phosphate dehydrogenase
Glut1: Glucose transporter-1
HBP: Hexosamine biosynthetic pathway
HK-2: Hexokinase-2
LDH: Lactate dehydrogenase
LDHA: Lactate dehydrogenase A
mtDNA: mitochondrial DNA
MUC1: Mucin1
NADPH: Nicotinamide adenine dinucleotide phosphate
NSCLC: Non-small cell lung cancer
PDK1: Pyruvate dehydrogenase kinase 1
PFK: Phosphofructokinase
PFKFB3: 6-phosphofructo-2-kinase/fructose-2,6-bisphosphatase 3
PGAM: Phosphoglyceric acidmutase
PKM2: Pyruvate kinase-2
PPARδ: Peroxisome-proliferator-activated receptor δ
PPP: Pentose phosphate pathway
ROS: Reactive Oxygen Species
SLC1-A5: Solute carrier family 1 member 5
TCA: Tricarboxylic acid cycle
TME: Tumor microenvironment
